# Prognostic impact of gene mutations in myelodysplastic syndromes with ring sideroblasts

**DOI:** 10.1038/s41408-017-0016-9

**Published:** 2017-11-20

**Authors:** Iván Martín, Esperanza Such, Blanca Navarro, Eva Villamón, Ana Vicente, Elvira Mora, Laia Pedrola, Mariam Ibáñez, María López-Pavía, Mar Tormo, Alicia Serrano, Miguel Ángel Sanz, José Cervera, Guillermo Sanz

**Affiliations:** 1 Hematology Department, University Hospital La Fe, Fernando Abril Martorell Avenue, 106, Valencia, 46026 Spain; 2 Hematology Department, University Hospital Clínico, Blasco Ibáñez Avenue, 17, 46010 Valencia, Spain; 3 Genetics Unit, University Hospital La Fe, Fernando Abril Martorell Avenue, 102, Valencia, 46026 Spain

Myelodysplastic syndromes (MDS) are a heterogeneous group of myeloid neoplasms with extremely variable clinical outcome^1^. The latest update of the World Health Organization (WHO) classification (2016) has defined various MDS subtypes on the basis of dysplastic and cytopenic lineages, the prevalence of blasts, the percentage of ring sideroblasts (RS), and the presence of cytogenetic and genetic abnormalities. In this assessment, the category defined by ring sideroblasts, MDS-RS, is subdivided into cases with single-lineage dysplasia (MDS-RS-SLD) and cases with multilineage dysplasia (MDS-RS-MLD). Moreover, in the presence of cytopenias, dysplasia, and as few as 5% of RS, the MDS-RS category can be established by the identification of a mutation in the *SF3B1* gene^2^. Therefore, genetic data like *SF3B1* mutation provide diagnostic utility in MDS and probably also relevant prognosis information. In recent years, next-generation sequencing (NGS) studies in MDS have found new recurrently mutated genes^3, 4^. Thus, we have utilized these findings for a specific mutational analysis of the MDS-RS subgroup.

We studied 122 patients diagnosed of MDS-RS according to the 2016 WHO classification. From them, 80 patients (66%) had been diagnosed by morphology of MDS-RS-SLD and 42 patients (34%) corresponded to MDS-RS-MLD (Supplementary Table 1). The International Prognostic Scoring System (IPSS) was low for 105 patients (86%) and intermediate-1 for 17 patients (14%). According to the Revised IPSS (IPSS-R), 53 patients (43%) were classified as very low risk, 60 patients (50%) as low risk, 8 patients (6%) as intermediate risk, and only 1 patient (1%) as high risk (Supplementary Table 1). The median follow-up was 35 months (95% confidence interval, 6–204 months), and 5 patients (4%) progressed to acute myeloid leukemia (AML, Supplementary Table 2).

DNA samples were isolated from bone marrow samples at diagnosis and were obtained with written informed consent in accordance with the Declaration of Helsinki, and the approval of the internal review of Bioethics and Medical Research at the University Hospital La Fe. NGS was based on AmpliSeq chemistry and was performed on an Ion Proton instrument. A custom panel of 39 genes with 659 amplicons was designed and allowed a 98% detection rate for 5% variant frequency at positions with an average sequencing coverage from 1000× to 5000× (Supplementary Methods; Supplementary Table 3). Mutations of *CALR* exon 9, not included in the original panel, were analyzed by Sanger sequencing^5^. In the statistical analyses *P* < 0.05 values were considered as statistically significant.

With this approach, up to 97% patients (118 out of 122) have been found to have a somatic mutation in at least one gene (Supplementary Tables 4 and 5). As it was expected, the majority of cases carried *SF3B1* mutations (106/122, 86.9%), but other mutations were also detected: *TET2* (38/122, 31.1%); *DNMT3A* (21/122, 17.2%); *JAK2* (8/122, 6.6%); *SRSF2* (6/122, 4.9%); *SETBP1* (6/122, 4.9%); *EZH2* (5/122, 4.1%); and *ZRSR2* (5/122, 4.1%) genes (Fig. 1).

**Fig. 1:**
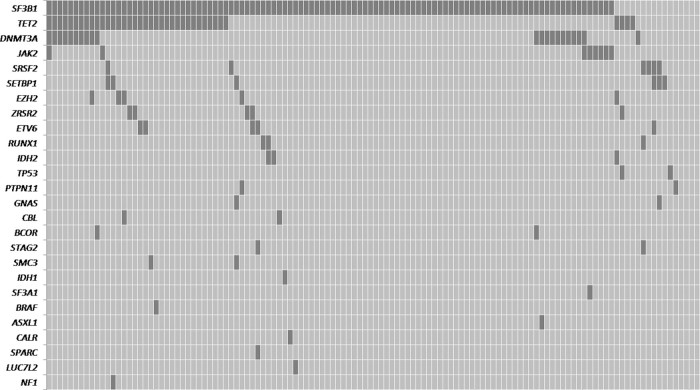
Distribution of genetic alterations of significantly mutated genes in 122 MDS-RS cases Each column represents an individual sample. Gray cells indicate a mutation in the gene described in that row on the left

In the overall series, patients carrying *SF3B1* mutations showed a lower proportion of poor prognosis chromosomal abnormalities compared with *SF3B1* ^ɯt^ cases (6% vs. 31%, *P*= 0.038; Supplementary Table 1). In addition, *SF3B1* mutations favorably influenced the overall survival (OS) of patients with single-lineage dysplasia (median OS, 88 vs. 22 months, *P* = 0.010; Supplementary Fig. 1A and B). Although in previous reports MDS-RS cases carrying *SF3B1* mutation appear to have a favorable prognosis compared to those without the mutation, the role of multilineage dysplasia vs. the *SF3B1* mutation remains controversial^6, 7^.

Of note, we found a significant positive correlation between the MDS-RS-MLD category and the median number of mutations per patient (2, range 0–5 vs. 1, range 0–5, for MDS-RS-MLD and MDS-RS-SLD; *P* = 0.003).

It was also observed that *SF3B1* ^mut^ patients with the K700E mutation showed a significant higher level of RS than *SF3B1* ^mut^ patients with other *SF3B1* mutations (median, 44% vs. 27%, *P* = 0.012). *SF3B1* mutations affect the gene expression of the iron transporter ABCB7 and determine the accumulation of aberrant mitochondrial ferritin in the erythroblasts^8^. It is likely that the distinct *SF3B1* mutations cause a different degree of ABCB7 downregulation and therefore a phenotype with more or less RS.

Mutations in other important splicing gene, *SRSF2*, were generally found in *SF3B1* ^ɯt^ patients suggesting that they would play a redundant role in disease pathogenesis^9^. Most *SRSF2* ^mut^ patients (83%) were clustered in lower-risk categories of the IPSS-R however showed a significantly lower platelet count (median, 93 × 10^9^ vs. 262 × 10^9^/L, *P* < 0.001) a higher red blood cell (RBC) transfusion-dependency (100% vs. 59%, *P* = 0.046), and an inferior OS (hazard ratio, HR = 10.89; *P* = 0.001) than *SRSF2* ^ɯt^ patients (Supplementary Tables 1 and 6, Supplementary Figure 1C).

In methylation categories, *TET2* mutations were found in a high number of MDS-RS patients although no differences in the clinical features were found according to their mutation status. Nevertheless, *TET2* ^mut^ patients showed a higher median number of mutations compared with *TET2* ^ɯt^ patients (3, range 0–5 vs. 1, range 0–5, *P* < 0.001). *TET2* mutations are currently considered as drivers of “clonal hematopoiesis of indeterminate potencial” and would lead to a primary permissive environment for subsequent genetic alterations^10^.

The other methylator gene frequently mutated was *DNMT3A*. Patients with *DNMT3A* mutations showed a more adverse clinical status with a significant higher RBC transfusion-dependency compared with *DNMT3A* ^ɯt^ patients (81% vs. 56%, *P* = 0.029; Supplementary Table 1). Furthermore, when *DNMT3A* mutations were considered according to their occurrence in the protein domain important differences were observed. In the regulatory domain (RG, exon 1–15, *n* = 9), frameshift and nonsense mutations predominantly occurred (80%; Fig. 2a) and were likely to be loss-of-function mutations and therefore would not exhibit significant changes in DNA methylation^11^. *DNMT3A_*RG mutations did not show any influence on OS or AML transformation. Conversely, in the DNMT3A methyltransferase domain (MT, exon 16–23, *n* = 12), missense mutations involving highly conserved residues were especially found (75%; Fig. 2a), suggesting that they may not be simple loss-of-function mutations and may confer a novel protein function^12, 13^. Several studies, especially based on R882 hotspot mutations, demonstrate profound loss of de novo methyltransferase activity resulting from the dominant negative consequences of the missense alterations. The mutant DNMT3A protein interacts with wild-type DNMT3A and DNMT3L proteins to form functionally deficient complexes that change the normal methylation patterns in the cell (Figure 2b)^12, 14^. Accordingly, the presence of *DNMT3A_*MT mutations in our series determined a more adverse clinical outcome with a very prominent RBC transfusion-dependence (92% vs. 57%, *P* = 0.017), a shorter OS, and a higher risk of AML progression (OS: HR = 4.99, *P* < 0.001; AML transformation: HR = 9.84, *P* = 0.047; Supplementary Table 6, Supplementary Fig. 1E and F).

**Fig. 2:**
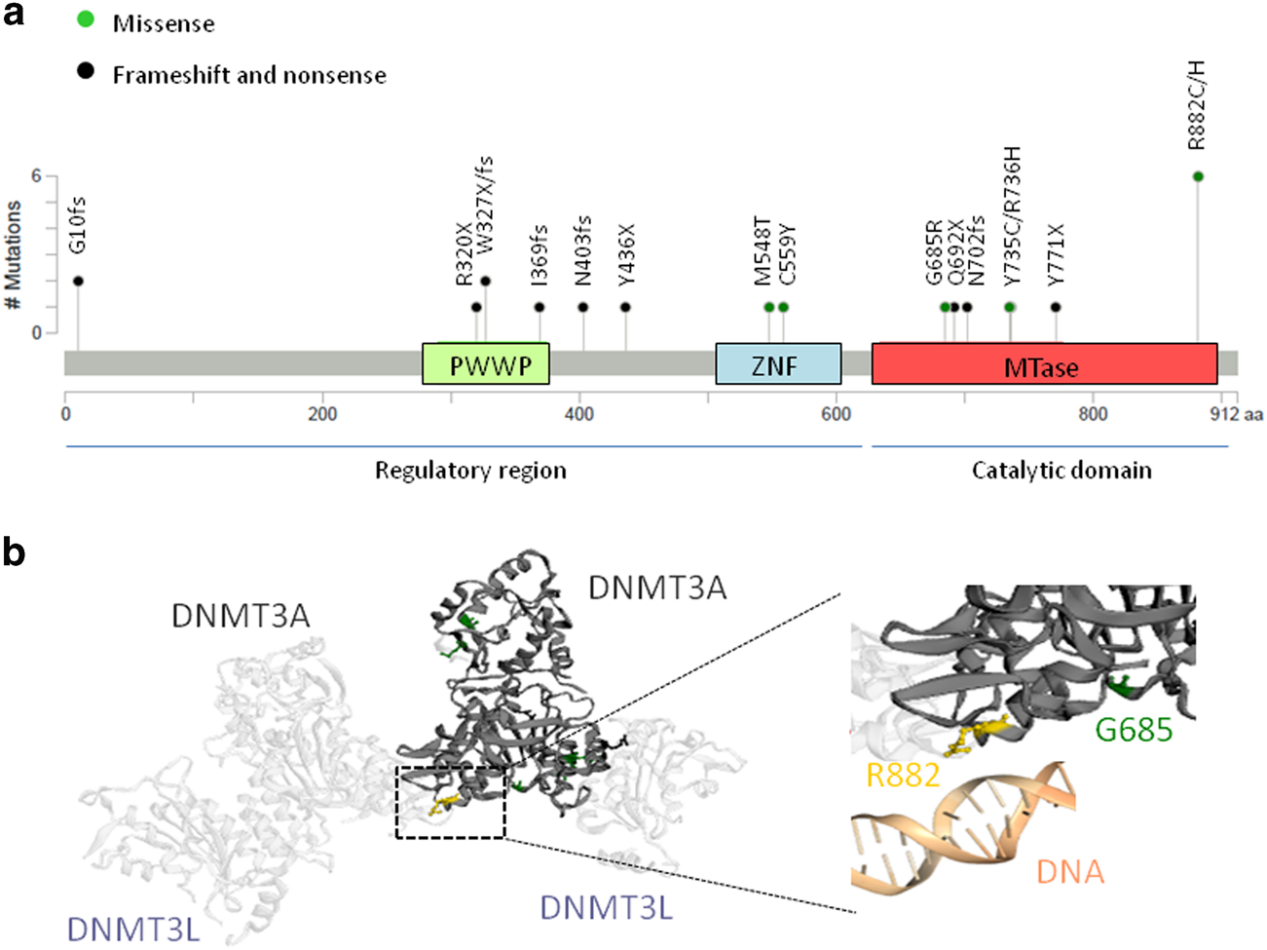
Locations of *DNMT3A* mutations and structure of DNMT3A protein Plot **a** reports the number of *DNMT3A* mutations found in the MDS-RS series and their locations with respect to the protein functional domain (MTase, methyltransferase; PWWP, chromatin targeting; ZNF, zinc finger). Plot **b** reports the interaction of two DNMT3L and two DNMT3A proteins to construct a tetramer with DNA methylating function. The R882 residue (in yellow color) is critical in the correct binding of the DNMT3A protein with the DNA molecule^12^

Finally, when analyzing other minority mutated genes, the negative clinical impact of the *EZH2* mutations should also be noted (OS, HR = 7.06, *P* = 0.004; Supplementary Table 6, Supplementary Fig. 1D). All *EZH2* ^mut^ patients were clustered in lower-risk categories of the IPSS and IPSS-R however had a high RBC transfusion-dependency at diagnosis and showed a median OS of 30 months, near to the OS observed in MDS patients within the intermediate IPSS-R risk category^15^.

In summary, the stratification of risk remains the essential step before treatment decision- making. Nevertheless, clinical behaviors differ from what expected on the basis of calculated prognostic indexes. In this context, our findings highlight the potential utility of *SF3B1*,* SRSF2*, *EZH2*, and *DNMT3A* gene mutations on prognostic risk stratification and treatment decisions in MDS-RS patients.

## Electronic supplementary material

Supplementary
