# Supplementary material for: Prognostic impact of gene mutations in myelodysplastic syndromes with ring sideroblasts
Source: Blood Cancer J. 2017 Nov 20;7(12):630. doi: 10.1038/s41408-017-0016-9 (PMC5802591; doi:10.1038/s41408-017-0016-9)
Supplement: Supplementary file 1 — Supplementary [file 41408_2017_16_MOESM1_ESM.pdf]

Prognostic impact of gene mutations in myelodysplastic syndromes with ring sideroblasts.

Supplementary methods, tables and figures in order of appearance.

Supplementary methods.

Primers design.

Primers were designed with Ion AmpliSeq™ technology (Thermo Fisher) to generate amplicons with an average length of 200 bp, providing a minimum coverage of 90% of the coding sequence. These primers were synthesized and pooled into 2 multiplex reactions based on PCR compatibility, minimizing the likelihood of primer-primer interactions.

Sequencing protocol.

For libraries preparation, 10 nanograms of each DNA sample was amplified using the gene panel Primer Pools and AmpliSeq HiFi mix (Thermo Fisher) in a Veriti® Thermal Cycler with 17 amplification cycles. PCR pools for each sample were subjected to primer digestion with the FuPa reagent (Thermo Fisher). Pooled amplicons were then ligated with universal adapters and different barcodes (Ion Xpress Barcodes™) for each sample. After a first purification with Agencourt® AMPure® XP Kit (Beckman Coulter), libraries were amplified with Platinum® PCR SuperMix High Fidelity and Library Amplification Primer Mix (Thermo Fisher). After a second purification, libraries were quantified using a Qubit® 2.0 Fluorometer with the Qubit® dsDNA HS Assay Kit and then they were normalized to 100 pmol/L. The normalized libraries were pooled in equal ratios for emulsion PCR (ePCR) on an Ion OneTouch System. Then, the templated Ion Sphere particles were enriched using the Ion OneTouch ES. The template-positive Ion PI Ion Sphere particles were loaded in an Ion PI chip V3 and sequenced on the Ion Proton instrument. Analyses were run on Torrent Suite and Ion Reporter Software providing sequence reads and variants in an exportable BAM, VCF and Excel file format.

Molecular analysis.

All listed variants were visually revised with the Integrative Genomics Viewer (IGV) software.[1] The literature and the Catalogue Of Somatic Mutations In Cancer database (COSMIC) were employed to explore the impact of somatic mutations found. The potential severity of *de novo* mutations was evaluated using the SIFT, Polyphen2 and Mutation Taster algorithms.[2] For variants located in splicing sites the Human Splicing Finder, NNSplice and NetGene2 algorithms were used to predict their pathogenicity.[3] Finally, the “cBioPortal Tools” were applied to construct the *DNMT3A* mutations map and the DNMT3A protein 3D structure.[4,5]

Statistical analysis.

Numerical variables were summarized by median and range and categorical variables described with count and relative frequency (percentage) of subjects in each category. Comparison of numerical variables between groups was performed using a nonparametric approach (Mann-Whitney test). Comparison of the distribution of categorical variables in different groups was performed with the X^2 test and Fisher’s exact test where appropriate. Overall survival was measured from the time of diagnosis to the last follow-up or death from any cause. Time to AML progression was measured from the date of MDS diagnosis to the time of AML diagnosis. Survival curves were generated using the Kaplan-Meier method and differences were assessed by log-rank test. For multivariable analysis, a Cox proportional hazard model was constructed. Statistical analyses were generated using the SPSS software and $P<0.05$ values were considered as statistically significant.

Supplementary Table 1. Clinical characteristics of MDS-RS patients and univariable analyses according to *SF3B1, SRSF2* and *DNMT3A* mutation status.

Characteristics	All	*SF3B1* Wild-type	*SF3B1* Mutated	*P* value	*SRSF2* Wild-type	*SRSF2* Mutated	*P* value	*DNMT3A* Wild-type	*DNMT3A* Mutated	*P* value
**Total (MDS-RS), no (%)**	122	16 (13)	106 (87)		116 (95)	6 (5)		101 (83)	21 (17)	
**Age**				0.42			0.32			0.09
Median	75	74	75		75	83		76	72	
Range	31-90	31-90	38-88		31-90	72-84		38-90	31-85	
**Sex, no (%)**				0.59			0.24			**0.021**
Male	68 (56)	9 (56)	59 (56)		66 (57)	2 (33)		61 (60)	7 (33)	
Female	54 (44)	7 (44)	47 (44)		50 (43)	4 (67)		40 (40)	14 (67)	
**WHO 2016 subtype, no (%)**				0.28			0.66			0.56
MDS-RS-SLD	80 (66)	9 (56)	71 (67)		76 (66)	4 (67)		66 (65)	14 (67)	
MDS-RS-MLD	42 (34)	7 (44)	35 (33)		40 (34)	2 (33)		35 (35)	7 (33)	
**Karyotype risk, no (%)**				**0.038**			0.66			0.75
Low	111 (91)	11 (69)	100(94)		106 (91)	5 (83)		92 (91)	19 (91)	
Intermediate	9 (7)	4 (25)	5 (5)		8 (7)	1 (17)		7 (7)	2 (9)	
High	2 (2)	1 (6)	1 (1)		2 (2)	0 (0)		2 (2)	0 (0)	
**Hemoglobin (g/dl)**				0.55			0.63			0.57
Median	9.9	9.4	9.9		9.9	9.7		9.9	9.7	
Range	5-14	7-14	5-13		5-14	9-12		5-14	8-12	
**WBC count (×10⁹/L)**				0.30			0.50			0.17
Median	6.0	4.4	6.0		6.0	4.3		6.2	5.2	
Range	2-16	2-16	2-14		2-16	3-7		2-16	4-14	
**PMN (×10⁹/L)**				0.16			0.56			0.40
Median	58	58	57		57	63		58	57	
Range	14-82	14-76	20-82		14-82	53-65		14-82	20-72	
**Platelet count (×10⁹/L)**				**<0.001**			**<0.001**			0.17
Median	253	146	267		262	93		252	248	
Range	18-486	18-284	84-486		18-486	37-129		18-486	110-389	
**BM blasts (%)**				0.13			0.41			0.27
Median	1	1	1		1	1		1	1	
Range	0-4	0-4	0-4		0-4	0-2		0-4	0-3	
**BM ring sideroblasts (%)**				0.51			0.40			0.37
Median	39	37	40		40	38		37	41	
Range	15-95	15-95	15-95		15-95	20-59		15-95	20-95	
**IPSS, no (%)**				**0.002**			**0.035**			0.41
Low risk	105 (86)	9 (56)	96 (91)		102 (88)	3 (50)		86 (85)	19 (91)	
Intermediate-1	17 (14)	7 (44)	10 (9)		14 (12)	3 (50)		15 (15)	2 (9)	
**IPSS-R, no (%)**				**0.005**			0.64			0.69
Very low risk	53 (43)	3 (19)	50 (47)		51 (44)	2 (33)		45 (44)	8 (38)	
Low risk	60 (50)	9 (56)	51 (48)		57 (49)	3 (50)		48 (48)	12 (57)	
Intermediate	8 (6)	3 (19)	5 (5)		7 (6)	1 (17)		7 (7)	1 (5)	
High	1 (1)	1 (6)	0 (0)		1 (1)	0 (0)		1 (1)	0 (0)	
**Transfusion dependence, no (%)**				0.27			**0.046**			**0.029**
Yes	74 (61)	12 (75)	62 (58)		68 (59)	6 (100)		57 (56)	17 (81)	
No	48 (39)	4 (25)	44 (42)		48 (41)	0 (0)		44 (44)	4 (19)	

MDS-RS-SLD: myelodysplastic syndrome with ring sideroblasts and single lineage dysplasia; MDS-RS-MLD: myelodysplastic syndrome with ring sideroblasts and multilineage dysplasia; WBC: white blood count; PMN: polymorphonuclear leukocytes; BM: bone marrow; IPSS: International Prognostic Scoring System; IPSS-R: Revised International Prognostic Scoring System

Supplementary Table 2. Main characteristics of MDS-RS patients with AML progression.

Patient	Age	WHO	IPSS	IPSS-R	Karyotype	*SF3B1*	*DNMT3A*	Other mutations	Years to AML	Subtype AML	Survival (years), status
MDS-RS_7	80	MDS-RS-MLD	Low	Low	46,XX	K700E	No	No	7.8	M0	8.0 (d)
MDS-RS_13	65	MDS-RS-SLD	Low	Low	46,XY	K700E	R882H	*TET2* (F1285del)	3.9	M1	4.8 (d)
MDS-RS_74	76	MDS-RS-MLD	Low	Int	46,XY, del(1)(?q23)	K700E	No	*TET2* (L1212fs)	0.9	M4	2.1 (d)
MDS-RS_59	64	MDS-RS-MLD	Low	Low	46,XY	K700E	R882C	*BCOR* (V598fs)	0.9	M2	1.7 (d)
MDS-RS_105	75	MDS-RS-SLD	Low	Low	46,XY	No	No	*EZH2* (D657Y), *IDH2* (R140Q)	2.3	M1	2.5 (d)

MDS-RS-SLD: myelodysplastic syndrome with ring sideroblasts and single lineage dysplasia; MDS-RS-MLD: myelodysplastic syndrome with ring sideroblasts and multilineage dysplasia; IPSS: International Prognostic Scoring System; IPSS-R: Revised International Prognostic Scoring System; int: intermediate; K: lysine; E: glutamic acid; R: arginine; H: histidine; C: cysteine; F: phenylalanine; L: leucine; V: valine; D: aspartic acid; Y: tyrosine; Q: glutamine; AML: acute myeloid leukemia; status, d: dead; a: alive.

Supplementary Table 3. Core panel of sequenced genes.

Gene symbol	Ensembl gene Id.	Transcript id.	Exons
ASXL1	ENSG00000171456	NM_015338.5	8-11
BCOR	ENSG00000183337	NM_001123385.1	all
BRAF	ENSG00000157764	NM_004333.4	all
CBL	ENSG00000110395	NM_005188.3	8,9
CDKN2A	ENSG00000147889	NM_000077.4	all
CEBPA	ENSG00000245848	NM_004364.4	all
DNMT3A	ENSG00000119772	NM_022552.4	all
ETV6	ENSG00000139083	NM_001987.4	all
EZH2	ENSG00000106462	NM_004456.4	all
FLT3	ENSG00000122025	NM_004119.2	13-15, 20
GNAS	ENSG00000087460	NM_080425.2	all
IDH1	ENSG00000138413	NM_005896.3	3,4
IDH2	ENSG00000182054	NM_002168.2	4
JAK2	ENSG00000096968	NM_004972.3	11-15
KIT	ENSG00000157404	NM_000222.2	2, 8-12, 17
KRAS	ENSG00000133703	NM_033360.3	2-4
LUC7L2	ENSG00000146963	NM_001244585.1	all
MPL	ENSG00000117400	NM_005373	10
NF1	ENSG00000196712	NM_001042492.2	all
NPM1	ENSG00000181163	NM_002520.6	10
NRAS	ENSG00000213281	NM_002524.4	1-4
PHF6	ENSG00000156531	NM_032458.2	all
PTPN11	ENSG00000179295	NM_002834.3	all
RAD21	ENSG00000164754	NM_006265.2	all
RPS14	ENSG00000164587	NM_001025070.1	2-5
RUNX1	ENSG00000159216	NM_001754.4	all
SETBP1	ENSG00000152217	NM_015559.2	4
SF1	ENSG00000168066	NM_001178030.1	all
SF3A1	ENSG00000099995	NM_005877.4	all
SF3B1	ENSG00000115524	NM_012433.2	10-16
SMC3	ENSG00000108055	NM_005445.3	all
SPARC	ENSG00000113140	NM_003118.3	all
SRSF2	ENSG00000161547	NM_003016.4	1
STAG2	ENSG00000101972	NM_001042749.2	all
TET2	ENSG00000168769	NM_001127208.2	all
TP53	ENSG00000141510	NM_001276760.1	3-11
U2AF1	ENSG00000160201	NM_006758	1-6
WT1	ENSG00000184937	NM_024426.4	6-10
ZRSR2	ENSG00000169249	NM_005089.3	all

Supplementary Table 4. Missense, frameshift and nonsense mutations in study.

SAMPLE ID.	GENE	PROTEIN	CODING	SIFT	POLYPHEN	MUTATION TASTER	COSMIC	ANNOTATION
MDS-RS_1	*SF3B1*	p.Lys700Glu	c.2098A>G	0	1	damage	84677	Oncogenic
	SF3B1	p.Lys666Arg	c.1997A>G	0	1	damage	131553	Oncogenic
MDS-RS_2	*SF3B1*	p.Asn626Asp	c.1876A>G	0	1	damage	131555	Oncogenic
	TET2	p.Lys1173fs	c.3516_3523del			damage		Oncogenic
	TET2	p.Cys1273Phe	c.3818G>T	0	1	damage	87135	Oncogenic
	EZH2	p.Lys740Ter	c.2218A>T			damage		Oncogenic
MDS-RS_3	*SF3B1*	p.Gly742Asp	c.2225G>A	0.08	0.966	damage	145923	Oncogenic
MDS-RS_4	*DNMT3A*	p.Tyr735Cys	c.2204A>G	0	1	damage	133126	Oncogenic
	SF3B1	p.Lys700Glu	c.2098A>G	0	1	damage	84677	Oncogenic
MDS-RS_5	*SF3B1*	p.Gly742Asp	c.2225G>A	0.08	0.966	damage	145923	Oncogenic
	SRSF2	p.Pro95Arg	c.284C>G	0.05	0.134	damage	211661	Oncogenic
	TET2	p.Asp1427Val	c.4280A>T	0	1	damage		Oncogenic
	SETBP1	p.His1100Arg	c.3299A>G	0	0.998	damage		Oncogenic
MDS-RS_6	*SF3B1*	p.Lys700Glu	c.2098A>G	0	1	damage	84677	Oncogenic
MDS-RS_7	*SF3B1*	p.Lys700Glu	c.2098A>G	0	1	damage	84677	Oncogenic
MDS-RS_8	*SF3B1*	p.His662Gln	c.1986C>A	0	1	damage	130416	Oncogenic
	JAK2	p.Val617Phe	c.1849G>T	0	0.996	damage	12600	Oncogenic
	SF3A1	p.Arg587Cys	c.1759C>T	0	1	damage		Oncogenic
MDS-RS_9	*SF3B1*	p.Lys700Glu	c.2098A>G	0	1	damage	84677	Oncogenic
MDS-RS_10	*SF3B1*	p.Lys700Glu	c.2098A>G	0	1	damage	84677	Oncogenic
MDS-RS_11	*SF3B1*	p.Lys666Thr	c.1997A>C	0	1	damage	131556	Oncogenic
	JAK2	p.Val617Phe	c.1849G>T	0	0.996	damage	12600	Oncogenic
MDS-RS_12	*SF3B1*	p.Lys700Glu	c.2098A>G	0	1	damage	84677	Oncogenic
MDS-RS_13	*DNMT3A*	p.Arg882His	c.2645G>A	0	1	damage	52944	Oncogenic
	SF3B1	p.Lys700Glu	c.2098A>G	0	1	damage	84677	Oncogenic
	TET2	p.Phe1285del	c.3852_3854del			damage	211724	Oncogenic
MDS-RS_14	*SF3B1*	p.Lys700Glu	c.2098A>G	0	1	damage	84677	Oncogenic
MDS-RS_15	*SF3B1*	p.Glu622Asp	c.1866G>C	0	1	damage	132938	Oncogenic
MDS-RS_16	*TP53*	p.Arg209Gln	c.626G>A	0	0.998	damage		Oncogenic
MDS-RS_17	*SF3B1*	p.Lys700Glu	c.2098A>G	0	1	damage	84677	Oncogenic
	TET2	p.Thr246fs	c.737_737del			damage		Oncogenic
	BRAF	p.Ile710fs	c.2127_2128ins			damage		Oncogenic
MDS-RS_18	*DNMT3A*	p.Met548Thr	c.1643T>C	0	1	damage		Oncogenic
	SF3B1	p.Lys700Glu	c.2098A>G	0	1	damage	84677	Oncogenic
	SF3B1	p.His662Tyr	c.1984C>T	0	1	damage	131560	Oncogenic
	TET2	p.Met1729fs	c.5170_5171ins			damage		Oncogenic
	EZH2	p.Asn673Ile	c.2018A>T	0	1	damage		Oncogenic
MDS-RS_19	*DNMT3A*	p.Arg882Cys	c.2644C>T	0	1	damage	53042	Oncogenic
	SF3B1	p.His662Gln	c.1986C>G	0	1	damage	130416	Oncogenic
	JAK2	p.Val617Phe	c.1849G>T	0	0.996	damage	12600	Oncogenic
MDS-RS_20	*PTPN11*	p.Gln587Glu	c.1759C>G	0.57	0.064	damage		Possible oncogenic
MDS-RS_21	*SF3B1*	p.Lys700Glu	c.2098A>G	0	1	damage	84677	Oncogenic
	TET2	p.Gly1288Ser	c.3862G>A	0	1	damage	110780	Oncogenic
MDS-RS_22								
MDS-RS_23	*DNMT3A*	p.Arg771Ter	c.2311C>T			damage	231563	Oncogenic
	SF3B1	p.His662Gln	c.1986C>A	0	1	damage	130416	Oncogenic
	TET2	p.Arg1202Ile	c.3605G>T	0	1	damage		Oncogenic
MDS-RS_24	*SF3B1*	p.Lys700Glu	c.2098A>G	0	1	damage	84677	Oncogenic
MDS-RS_25	*SF3B1*	p.Arg625Gly	c.1873C>G	0	1	damage	1169490	Oncogenic
MDS-RS_26	*SF3B1*	p.Lys700Glu	c.2098A>G	0	1	damage	84677	Oncogenic
	TET2	p.Tyr867His	c.2599T>C	0	0.999	damage	327337	Oncogenic
MDS-RS_27								

	SF3B1	p.Glu622Asp	c.1866G>T	0	1	damage	132938	Oncogenic
**MDS-RS_28**	*EZH2*	p.Tyr133Asp	c.397T>G	0	0.068	damage	144172	Oncogenic
	PTPN11	p.Gly503Ala	c.1508G>C	0	0.998	damage	13027	Oncogenic
**MDS-RS_29**	*SF3B1*	p.Lys700Glu	c.2098A>G	0	1	damage	84677	Oncogenic
**MDS-RS_30**	*SF3B1*	p.Lys666Gln	c.1996A>C	0	1	damage	132950	Oncogenic
	DNMT3A	p.Trp327fs	c.979_979del			damage		Oncogenic
**MDS-RS_31**	*SF3B1*	p.Lys700Glu	c.2098A>G	0	1	damage	84677	Oncogenic
	ASXL1	p.Gln283His	c.849G>C	0.02	1	damage		Oncogenic
**MDS-RS_32**	*SF3B1*	p.Glu622Asp	c.1866G>T	0	1	damage	132938	Oncogenic
	SF3B1	p.Lys700Glu	c.2098A>G	0	1	damage	84677	Oncogenic
**MDS-RS_33**	*LUC7L2*	p.Glu111Asp	c.333G>C	0.03	0.565	damage		Possible oncogenic
	TET2	p.Gln1541Ter	c.4621C>T			damage		Oncogenic
**MDS-RS_34**	*DNMT3A*	p.Gly10fs	c.27_28ins			damage		Oncogenic
	SF3B1	p.Lys700Glu	c.2098A>G	0	1	damage	84677	Oncogenic
	SF3B1	p.Lys700Glu	c.2098A>G	0	1	damage	84677	Oncogenic
**MDS-RS_35**	*ZRSR2*	p.Gln32Ter	c.94C>T			damage		Oncogenic
	ZRSR2	p.Cys312Tyr	c.935G>A	0	1	damage		Oncogenic
	DNMT3A	p.Arg736His	c.2207G>A	0.39	0.997	damage	133737	Oncogenic
	SF3B1	p.Lys700Glu	c.2098A>G	0	1	damage	84677	Oncogenic
**MDS-RS_36**	*SF3B1*	p.His662Asp	c.1984C>G	0	1	damage	131560	Oncogenic
	TET2	p.Gln80Ter	c.238C>T			damage	43428	Oncogenic
	TET2	p.Gln969Ter	c.2905C>T			damage		Oncogenic
	SRSF2	p.Pro95His	c.284C>A	0	1	damage	211504	Oncogenic
**MDS-RS_37**	*SETBP1*	p.Asp868Asn	c.2602G>A	0	1	damage	1318400	Oncogenic
	GNAS	p.Arg844Cys	c.2530C>T	0	1	damage	1566192	Oncogenic
**MDS-RS_38**								
**MDS-RS_39**	*SF3B1*	p.Lys700Glu	c.2098A>G	0	1	damage	84677	Oncogenic
**MDS-RS_40**	*SF3B1*	p.Lys700Glu	c.2098A>G	0	1	damage	84677	Oncogenic
**MDS-RS_41**	*SF3B1*	p.Gly742Asp	c.2225G>A	0	1	damage	145923	Oncogenic
	SF3B1	p.Arg625Gly	c.1873C>G	0	1	damage	1169490	Oncogenic
**MDS-RS_42**	*SF3B1*	p.His662Gln	c.1986C>A	0	1	damage	130416	Oncogenic
	JAK2	p.Val617Phe	c.1849G>T	0	0.996	damage	12600	Oncogenic
**MDS-RS_43**	*SF3B1*	p.Lys700Glu	c.2098A>G	0	1	damage	84677	Oncogenic
**MDS-RS_44**	*SF3B1*	p.Lys666Asn	c.1998G>C	0	1	damage	131557	Oncogenic
	SRSF2	p.Pro95His	c.284C>A	0	0.065	damage	211504	Oncogenic
**MDS-RS_45**	*SF3B1*	p.Lys700Glu	c.2098A>G	0	1	damage	84677	Oncogenic
**MDS-RS_46**	*SF3B1*	p.Lys700Glu	c.2098A>G	0	1	damage	84677	Oncogenic
**MDS-RS_47**	*SF3B1*	p.Lys700Glu	c.2098A>G	0	1	damage	84677	Oncogenic
**MDS-RS_48**	*SF3B1*	p.Lys700Glu	c.2098A>G	0	1	damage	84677	Oncogenic
	CBL	p.Arg462Ter	c.1384C>T			damage	34079	Oncogenic
**MDS-RS_49**	*DNMT3A*	p.Arg882Cys	c.2644C>T	0	1	damage	53042	Oncogenic
	SF3B1	p.Lys700Glu	c.2098A>G	0	1	damage	84677	Oncogenic
**MDS-RS_50**	*DNMT3A*	p.Arg882His	c.2645G>A	0	1	damage	452944	Oncogenic
	SF3B1	p.Lys700Glu	c.2098A>G	0	1	damage	84677	Oncogenic
	SF3B1	p.Lys700Glu	c.2098A>G	0	1	damage	84677	Oncogenic
**MDS-RS_51**	*TET2*	p.His1219Tyr	c.3655C>T	0	1	damage		Oncogenic
	DNMT3A	p.Arg882Cys	c.2644C>T	0	1	damage	53042	Oncogenic
	DNMT3A	p.Gly685Arg	c.2053G>A	0.01	1	damage	1235163	Oncogenic
**MDS-RS_52**	*SF3B1*	p.Lys700Glu	c.2098A>G	0	1	damage	84677	Oncogenic
	TET2	p.Leu699Ter	c.2096T>A			damage		Oncogenic
	JAK2	p.Val617Phe	c.1849G>T	0	0.996	damage	12600	Oncogenic
**MDS-RS_53**	*DNMT3A*	p.Asn403fs	c.1208_1208del			damage		Oncogenic
	SF3B1	p.Arg625Leu	c.1874G>T	0	1	damage	110695	Oncogenic
	DNMT3A	p.Ile369fs	c.1105_1117del			damage	144493	Oncogenic
**MDS-RS_54**	*SF3B1*	p.Glu622Asp	c.1866G>T	0	1	damage	132938	Oncogenic
	TET2	p.Thr1884Ala	c.5650A>G	0	1	damage		Oncogenic
	BCOR	p.Ala970Glu	c.2909C>A	0	1	damage		Oncogenic

**MDS-RS_55**	*DNMT3A*	p.Asp702fs	c.2104_2104del			damage		Oncogenic
	DNMT3A	p.Gly10fs	c.27_28ins			damage		Oncogenic
	SF3B1	p.Lys666Thr	c.1997A>C	0	1	damage	131556	Oncogenic
**MDS-RS_56**								
**MDS-RS_57**	*SF3B1*	p.Lys666Gln	c.1996A>C	0	1	damage	132950	Oncogenic
	TET2	p.Glu28fs	c.81_82del			damage	1426202	Oncogenic
	TET2	p.Cys677fs	c.2027_2030del			damage	43519	Oncogenic
**MDS-RS_58**	*SF3B1*	p.His662Gln	c.1986C>A	0	1	damage	130416	Oncogenic
	TET2	p.Gln1030Ter	c.3088C>T			damage	96929	Oncogenic
	TET2	p.His1219Asn	c.3655C>A	0	1	damage		Oncogenic
	TET2	p.Arg1891Gly	c.5671A>G	0	1	damage		Oncogenic
	ZRSR2	p.Trp111Ter	c.333G>A			damage		Oncogenic
**MDS-RS_59**	*DNMT3A*	p.Arg882Cys	c.2644C>T	0	1	damage	53042	Oncogenic
	SF3B1	p.Lys700Glu	c.2098A>G	0	1	damage	84677	Oncogenic
	BCOR	p.Val598fs	c.1791_1792ins			damage		Oncogenic
**MDS-RS_60**	*SF3B1*	p.Lys666Arg	c.1997A>G	0	1	damage	131553	Oncogenic
	RUNX1	p.Arg169fs	c.506_507ins			damage	1318812	Oncogenic
**MDS-RS_61**	*SF3B1*	p.Lys700Glu	c.2098A>G	0	1	damage	84677	Oncogenic
	CALR	p.Glu380Gly	c.1139A>G	0.4	0	damage		Possible oncogenic
**MDS-RS_62**	*SF3B1*	p.Lys700Glu	c.2098A>G	0	1	damage	84677	Oncogenic
	SPARC	p.Arg268His	c.803G>A	0.06	1	damage		Oncogenic
	ETV6	p.Met389Val	c.1165A>G	0	1	damage		Oncogenic
	STAG2	p.Arg1012Gln	c.3035G>A	0.15	0.971	damage	1465378	Oncogenic
**MDS-RS_63**	*SF3B1*	p.Lys666Arg	c.1997A>G	0	1	damage	131553	Oncogenic
**MDS-RS_64**	*SF3B1*	p.Glu622Asp	c.1866G>C	0	1	damage	132938	Oncogenic
**MDS-RS_65**	*SF3B1*	p.His662Gln	c.1986C>G	0	1	damage	130416	Oncogenic
	TET2	p.Gly1152Glu	c.3455G>A	0	1	damage	53268	Oncogenic
**MDS-RS_66**	*SF3B1*	p.Asn626Asp	c.1876A>G	0	1	damage	131555	Oncogenic
	TET2	p.Gln866fs	c.2593_2597del			damage		Oncogenic
**MDS-RS_67**	*DNMT3A*	p.Arg320Ter	c.958C>T			damage	133721	Oncogenic
	SF3B1	p.Lys700Glu	c.2098A>G	0	1	damage	84677	Oncogenic
	TET2	p.Phe1309Leu	c.3927T>A	0	0.999	damage		Oncogenic
**MDS-RS_68**	*SF3B1*	p.Lys700Glu	c.2098A>G	0	1	damage	84677	Oncogenic
	TET2	p.Pro1419Arg	c.4256C>G	0	1	damage	100055	Oncogenic
	JAK2	p.Val617Phe	c.1849G>T	0	0.996	damage	12600	Oncogenic
**MDS-RS_69**	*SF3B1*	p.Lys700Glu	c.2098A>G	0	1	damage	84677	Oncogenic
**MDS-RS_70**	*SF3B1*	p.Lys700Glu	c.2098A>G	0	1	damage	84677	Oncogenic
	TET2	p.Gln1034Ter	c.3100C>T			damage		Oncogenic
**MDS-RS_71**	*SETBP1*	p.Asp868Asn	c.2602G>A	0	1	damage	1318400	Oncogenic
	SETBP1	p.Gly870Ser	c.2608G>A	0	1	damage	1234973	Oncogenic
**MDS-RS_72**	*SF3B1*	p.Arg625Leu	c.1874G>T	0	1	damage	110695	Oncogenic
**MDS-RS_73**	*TET2*	p.Gln591Ter	c.1771C>T			damage		Oncogenic
	TET2	p.His1416Asp	c.4246C>G	0	1	damage		Oncogenic
**MDS-RS_74**	*SF3B1*	p.Lys700Glu	c.2098A>G	0	1	damage	84677	Oncogenic
	TET2	p.Leu1212fs	c.3633_3633del			damage	87124	Oncogenic
**MDS-RS_75**	*ETV6*	p.Tyr346Cys	c.1037A>G	0	1	damage		Oncogenic
	SRSF2	p.Pro95_Arg102del	c.284_307del			damage	146289	Oncogenic
	SETBP1	p.Asp868Asn	c.2602G>A	0	1	damage	1318400	Oncogenic
	SETBP1	p.Asp874Asn	c.2620G>A	0	1	damage	1717365	Oncogenic
**MDS-RS_76**	*SF3B1*	p.Lys700Glu	c.2098A>G	0	1	damage	84677	Oncogenic
	ETV6	p.Leu201Pro	c.602T>C	0.01	0.641	damage	546746	Oncogenic
	TET2	p.Glu783Ter	c.2347G>T			damage		Oncogenic
**MDS-RS_77**	*DNMT3A*	p.Gln692Ter	c.2074C>T			damage		Oncogenic
	SF3B1	p.Thr663Ile	c.1988C>T	0	1	damage	145921	Oncogenic
	TET2	p.Gln1084Pro	c.3251A>C	0	0.325	damage		Oncogenic
**MDS-RS_78**	*TET2*	p.Glu783Ter	c.2347G>T			damage		Oncogenic
	ETV6	p.Leu201Pro	c.602T>C	0.01	0.641	damage	546746	Oncogenic

**MDS-RS_79**	*SF3B1*	p.Lys700Glu	c.2098A>G	0	1	damage	84677	Oncogenic
	TET2	p.Cys1289Tyr	c.3866G>A	0	1	damage	87136	Oncogenic
**MDS-RS_80**	*SF3B1*	p.Lys700Glu	c.2098A>G	0	1	damage	84677	Oncogenic
	TET2	p.Gln769fs	c.2305_2305del			damage	87100	Oncogenic
**MDS-RS_81**	*SRSF2*	p.Pro95_Arg102del	c.284_307del			damage	146289	Oncogenic
	STAG2	p.Glu1023Val	c.3068A>T	0	0.974	damage		Oncogenic
**MDS-RS_82**	*SF3B1*	p.Lys700Glu	c.2098A>G	0	1	damage	84677	Oncogenic
**MDS-RS_83**	*SF3B1*	p.Lys700Glu	c.2098A>G	0	1	damage	84677	Oncogenic
**MDS-RS_84**	*SF3B1*	p.Lys700Glu	c.2098A>G	0	1	damage	84677	Oncogenic
**MDS-RS_85**	*SF3B1*	p.Lys700Glu	c.2098A>G	0	1	damage	84677	Oncogenic
**MDS-RS_86**	*TET2*	p.Gln876Ter	c.2626C>T			damage	211613	Oncogenic
	TP53	p.Cys176Trp	c.528C>G	0	1	damage	1268357	Oncogenic
	ZRSR2	p.Tyr292Asn	c.874T>A	0	1	damage		Oncogenic
**MDS-RS_87**	*SF3B1*	p.Lys666Arg	c.1997A>G	0	1	damage	131553	Oncogenic
**MDS-RS_88**	*SF3B1*	p.Lys700Glu	c.2098A>G	0	1	damage	84677	Oncogenic
	TET2	p.Asn1805fs	c.5413_5413del			damage		Oncogenic
	NF1	p.Thr1730Ser	c.5189C>G	0.01	1	damage		Oncogenic
	SETBP1	p.Arg1321His	c.3962G>A	0	1	damage		Oncogenic
**MDS-RS_89**	*SF3B1*	p.Glu622Asp	c.1866G>T	0	1	damage	132938	Oncogenic
	IDH2	p.Arg140Gln	c.419G>A	0	1	damage	41590	Oncogenic
**MDS-RS_90**	*SF3B1*	p.His662Gln	c.1986C>G	0	1	damage	130416	Oncogenic
**MDS-RS_91**	*SF3B1*	p.His662Asp	c.1984C>G	0	1	damage	131560	Oncogenic
	ETV6	p.Leu201Pro	c.602T>C	0.01	0.641	damage	546746	Oncogenic
	ZRSR2	p.Lys90Met	c.269A>T	0	0.998	damage		Oncogenic
**MDS-RS_92**	*SF3B1*	p.Lys666Thr	c.1997A>C	0	1	damage	131556	Oncogenic
	TET2	p.Leu878fs	c.2632_2632del			damage		Oncogenic
	SMC3	p.Tyr1140Asn	c.3418T>A	0	1	damage		Oncogenic
**MDS-RS_93**	*IDH1*	p.Phe32Val	c.94T>G	0.02	0.303	damage	1581043	Oncogenic
	SF3B1	p.Lys700Glu	c.2098A>G	0	1	damage	84677	Oncogenic
**MDS-RS_94**	*SF3B1*	p.Lys700Glu	c.2098A>G	0	1	damage	84677	Oncogenic
**MDS-RS_95**	*SF3B1*	p.Lys700Glu	c.2098A>G	0	1	damage	84677	Oncogenic
	SETBP1	p.Arg627Cys	c.1879C>T	0	1	damage	1236142	Oncogenic
	GNAS	p.Arg844His	c.2531G>A	0	1	damage	94388	Oncogenic
**MDS-RS_96**	*SF3B1*	p.Arg625Cys	c.1873C>T	0	1	damage	110696	Oncogenic
**MDS-RS_97**	*SF3B1*	p.Lys700Glu	c.2098A>G	0	1	damage	84677	Oncogenic
**MDS-RS_98**	*SF3B1*	p.Lys700Glu	c.2098A>G	0	1	damage	84677	Oncogenic
**MDS-RS_99**	*DNMT3A*	p.Cys559Tyr	c.1676G>A	0	1	damage		Oncogenic
	SF3B1	p.Lys700Glu	c.2098A>G	0	1	damage	84677	Oncogenic
	TET2	p.Gln341Ter	c.1021C>T	0	1	damage		Oncogenic
**MDS-RS_100**	*SF3B1*	p.Lys700Glu	c.2098A>G	0	1	damage	84677	Oncogenic
	JAK2	p.Pro708Ser	c.2122C>T	1	0.999	damage		Oncogenic
**MDS-RS_101**	*SF3B1*	p.Lys700Glu	c.2098A>G	0	1	damage	84677	Oncogenic
**MDS-RS_102**	*SF3B1*	p.Lys666Arg	c.1997A>G	0	1	damage	131553	Oncogenic
**MDS-RS_103**	*SF3B1*	p.Lys700Glu	c.2098A>G	0	1	damage	84677	Oncogenic
**MDS-RS_104**	*SF3B1*	p.Lys700Glu	c.2098A>G	0	1	damage	84677	Oncogenic
	TET2	p.Trp564Ter	c.1691G>A			damage	1716596	Oncogenic
	TET2	p.Ser714Ter	c.2141C>G			damage		Oncogenic
**MDS-RS_105**	*TET2*	p.Leu500fs	c.1494_1495ins			damage	211641	Oncogenic
	EZH2	p.Asp657Tyr	c.1969G>T	0	1	damage	1000720	Oncogenic
	IDH2	p.Arg140Gln	c.419G>A	0	1	damage	41590	Oncogenic
**MDS-RS_106**	*SRSF2*	p.Pro95Arg	c.284C>G	0.05	0.134	damage	211661	Oncogenic
**MDS-RS_107**	*SF3B1*	p.Lys666Asn	c.1998G>T	0	1	damage	131557	Oncogenic
**MDS-RS_108**	*SF3B1*	p.Lys700Glu	c.2098A>G	0	1	damage	84677	Oncogenic
**MDS-RS_109**	*TET2*	p.Gln684fs	c.2046_2046del			damage	211686	Oncogenic
**MDS-RS_110**	*SF3B1*	p.Trp658Arg	c.1972T>A	0	1	damage		Oncogenic
	TET2	p.Arg1404Ter	c.4210C>T			damage		Oncogenic
	CBL	p.Arg420Gln	c.1259G>A	0	1	damage	34077	Oncogenic

**MDS-RS_111**	*SF3B1*	p.Lys700Glu	c.2098A>G	0	1	damage	84677	Oncogenic
	TET2	p.Lys1339fs	c.4011_4012ins			damage	87145	Oncogenic
**MDS-RS_112**	*SF3B1*	p.Lys700Glu	c.2098A>G	0	1	damage	84677	Oncogenic
	TET2	p.Ser657Ter	c.1970C>G			damage		Oncogenic
	TET2	p.Gln1084Pro	c.3251A>C	0	0.325	damage		Oncogenic
**MDS-RS_113**	*SF3B1*	p.Asp584Glu	c.1752T>A	0.03	1	damage		Oncogenic
	IDH2	p.Arg140Gln	c.419G>A	0	1	damage	41590	Oncogenic
	RUNX1	p.Asn96Ser	c.287A>G	0.08	0.988	damage		Oncogenic
**MDS-RS_114**	*SF3B1*	p.Lys700Glu	c.2098A>G	0	1	damage	84677	Oncogenic
	TET2	p.Cys1271fs	c.3811_3812ins			damage	87134	Oncogenic
**MDS-RS_115**	*SF3B1*	p.Lys700Glu	c.2098A>G	0	1	damage	84677	Oncogenic
**MDS-RS_116**	*SF3B1*	p.Glu622Asp	c.1866G>C	0	1	damage	132938	Oncogenic
**MDS-RS_117**	*DNMT3A*	p.Trp327Ter	c.981G>A			damage		Oncogenic
	SF3B1	p.Lys700Glu	c.2098A>G	0	1	damage	84677	Oncogenic
**MDS-RS_118**	*SF3B1*	p.Lys700Glu	c.2098A>G	0	1	damage	84677	Oncogenic
**MDS-RS_119**	*SF3B1*	p.His662Gln	c.1986C>A	0	1	damage	130416	Oncogenic
	JAK2	p.Val617Phe	c.1849G>T	0	0.996	damage	12600	Oncogenic
**MDS-RS_120**	*SF3B1*	p.Lys700Glu	c.2098A>G	0	1	damage	84677	Oncogenic
**MDS-RS_121**	*DNMT3A*	p.Tyr436Ter	c.1308C>A			damage		Oncogenic
**MDS-RS_122**	*SF3B1*	p.Glu622Asp	c.1866G>T	0	1	damage	132938	Oncogenic

Supplementary Table 5. Splicing mutations in study.

SAMPLE ID.	LOCUS	REF/CHANGE	GENE	LOCATION	NNSPLICE	HSF	NETGENE2	MUTATION TASTER	ANNOTATION
**MDS-RS_55**	chr2:25458695	C/T	*DNMT3A*	splicesite_5	damage	damage	neutral	damage	Possible oncogenic
**MDS-RS_79**	chrX:15836766	G/A	*ZRSR2*	splicesite_3	damage	damage	damage	damage	Possible oncogenic
**MDS-RS_81**	chr21:36259138	A/G	*RUNX1*	splicesite_3	damage	damage	damage	damage	Possible oncogenic
**MDS-RS_95**	chr10:112342401	G/A	*SMC3*	splicesite_3	damage	neutral	damage	damage	Possible oncogenic
**MDS-RS_110**	chr7:148543690	T/TT	*EZH2*	splicesite_5	neutral	damage	neutral	damage	Possible oncogenic

Supplementary Table 6. Multivariable analyses for overall survival and risk of AML progression.

Variable	Overall survival			Risk of AML progression		
	HR	95% CI	*P* value	HR	95% CI	*P* value
**Model I**						
IPSS risk classification						
Intermediate-1 *vs.* Low	2.28	1.01 to 5.17	**0.048**	0.00		0.99
Age ≥75 *vs.* <75 years	4.47	2.13 to 9.39	**<0.001**	7.54	0.70 to 31.12	0.97
Sex	0.62	0.29 to 1.29	0.20	0.18	0.02 to 2.80	0.22
Transfusion dependence	1.38	0.59 to 3.26	0.46	1.23	0.41 to 3.60	0.76
ETV6 ^{mut} *vs.* *ETV6* ^{wt}	0.62	0.08 to 4.98	0.65	0.19		0.99
EZH2 ^{mut} *vs.* *EZH2* ^{wt}	6.86	1.79 to 21.32	**0.005**	13.86	0.22 to 36.28	0.39
JAK2 ^{mut} *vs.* *JAK2* ^{wt}	0.25	0.05 to 1.21	0.09	0.00		0.99
*DNMT3A_* MT ^{mut} *vs.* *DNMT3A_* MT ^{wt}	5.01	2.17 to 11.60	**<0.001**	7.92	1.22 to 24.86	**0.041**
*DNMT3A_* RG ^{mut} *vs.* *DNMT3A_* RG ^{wt}	1.16	0.32 to 4.29	0.82	0.00		0.99
TET2 ^{mut} *vs.* *TET2* ^{wt}	0.96	0.48 to 1.94	0.91	1.52	0.19 to 13.85	0.69
SF3B1 ^{mut} *vs.* *SF3B1* ^{wt}	1.29	0.49 to 3.34	0.60	1.26	0.03 to 28.23	0.91
SRSF2 ^{mut} *vs.* *SRSF2* ^{wt}	8.66	1.87 to 24.56	**0.006**	1.87		0.99
SETBP1 ^{mut} *vs.* *SETBP1* ^{wt}	1.82	0.38 to 8.73	0.46	0.00		0.99
ZRSR2 ^{mut} *vs.* *ZRSR2* ^{wt}	2.16	0.57 to 8.22	0.26	0.00		0.99
**Model II**						
IPSS-R risk classification						
Intermediate *vs.* Very Low	1.90	1.16 to 3.12	**0.011**	12.06	1.28 to 21.16	0.43
Intermediate *vs.* Low	1.36	0.84 to 4.23	0.18	14.59	1.28 to 26.15	0.09
Age ≥75 *vs.* <75 years	3.67	1.76 to 7.62	**0.001**	6.22	0.39 to 33.16	0.19
Sex	0.61	0.29 to 1.25	0.18	0.21	0.02 to 3.76	0.10
Transfusion dependence	1.41	0.61 to 3.26	0.42	1.42	0.72 to 4.12	0.84
ETV6 ^{mut} *vs.* *ETV6* ^{wt}	0.59	0.07 to 4.98	0.63	12.87		0.99
EZH2 ^{mut} *vs* . *EZH2* ^{wt}	7.06	1.87 to 22.42	**0.004**	16.23	0.77 to 40.25	0.12
JAK2 ^{mut} *vs.* *JAK2* ^{wt}	0.23	0.05 to 1.14	0.07	0.00		0.98
*DNMT3A_* MT ^{mut} *vs.* *DNMT3A_* MT ^{wt}	4.99	2.17 to 11.53	**<0.001**	9.84	1.13 to 27.73	**0.047**
*DNMT3A_* RG ^{mut} *vs.* *DNMT3A_* RG ^{wt}	0.90	0.25 to 3.29	0.88	0.00		0.99
TET2 ^{mut} *vs.* *TET2* ^{wt}	0.82	0.40 to 1.66	0.58	2.71	0.16 to 21.83	0.54
SF3B1 ^{mut} *vs.* *SF3B1* ^{wt}	1.65	0.63 to 4.30	0.30	7.48	0.14 to 26.15	0.29
SRSF2 ^{mut} *vs.* *SRSF2* ^{wt}	10.89	2.80 to 27.42	**0.001**	0.00		0.99
SETBP1 ^{mut} *vs.* *SETBP1* ^{wt}	1.74	0.33 to 9.12	0.51	0.00		0.99
ZRSR2 ^{mut} *vs.* *ZRSR2* ^{wt}	1.93	0.50 to 7.37	0.34	0.00		0.99

Cox proportional hazard regression models were constructed for the overall survival and for the risk of progression to acute myeloid leukemia (AML). These models were adjusted for the International Prognostic Scoring System (IPSS; Model I) and the Revised International Prognostic Scoring System (IPSS-R; Model II) risk classifications. Gene mutations (≥5 patients) were evaluated along with age (below *vs*. above median), sex, transfusion dependence and either IPSS or IPSS-R as candidates in Cox regression modelling. Abbreviation: HR, hazard ratio.

.

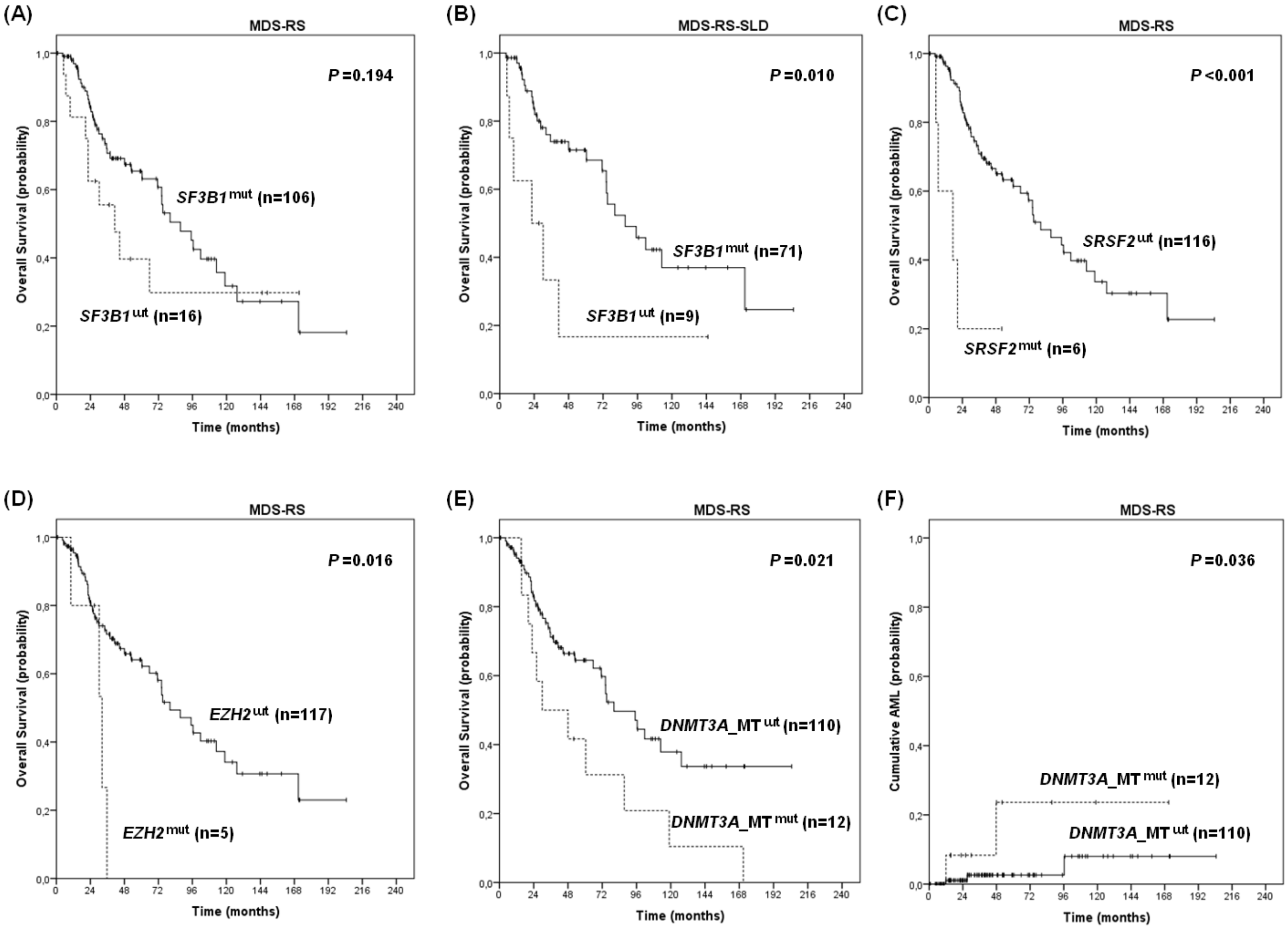

Supplementary Figure 1. Kaplan–Meier curves of MDS-RS patients according to their genetic alterations. Plots A and B report respectively overall survival (OS) according to *SF3B1* mutation status in the whole cohort and in the MDS-RS-SLD subgroup. Plots C and D report respectively OS according to *SRSF2* and *EZH2* mutation status. Plots E and F report respectively OS and risk of leukemic progression associated with *DNMT3A* mutations placed in methyltransferase domain.

Supplementary references.

1.- Thorvaldsdóttir H, Robinson JT, Mesirov JP. Integrative Genomics Viewer (IGV): high-performance genomics data visualization and exploration. *Brief Bioinform* 2013; **14**: 178-192.

2.- Grimm DG, Azencott CA, Aicheler F, Gieraths U, MacArthur DG, Samocha KE, *et al*. The evaluation of tools used to predict the impact of missense variants is hindered by two types of circularity. *Hum Mutat* 2015; **36**: 513-523.

3.- Jian X, Boerwinkle E, Liu X. In silico tools for splicing defect prediction: a survey from the viewpoint of end users. *Genet Med* 2014; **16**: 497-503.

4.- Cerami E, Gao J, Dogrusoz U, Gross BE, Sumer SO, Aksoy BA, *et al*. The cBio cancer genomics portal: an open platform for exploring multidimensional cancer genomics data. *Cancer Discov* 2012; **2**: 401-404.

5.- Gao J, Aksoy BA, Dogrusoz U, Dresdner G, Gross B, Sumer SO, *et al*. Integrative analysis of complex cancer genomics and clinical profiles using the cBioPortal. *Sci Signal* 2013; **6**: pl1.
